# The Multi-Modal Risk Analysis and Medical Prevention of Lumbar Degeneration, Fatigue, and Injury Based on FEM/BMD for Elderly Chinese Women Who Act as Stay-Home Grandchildren Sitters

**DOI:** 10.3389/fpubh.2021.700148

**Published:** 2021-11-19

**Authors:** Na Li, María José Cavagnaro, Kun Xiong, Xianping Du, Jian Shi

**Affiliations:** ^1^Department of Radiology, The Third Xiangya Hospital, Central South University, Changsha, China; ^2^College of Medicine-Phoenix, The University of Arizona, Phoenix, AZ, United States; ^3^Department of Anatomy and Neurobiology, School of Basic Medical Sciences, Central South University, Changsha, China; ^4^Department of Mechanical and Aerospace Engineering, Rutgers University, New Brunswick, NJ, United States; ^5^Department of Spine Surgery, The Third Xiangya Hospital, Central South University, Changsha, China

**Keywords:** reverse engineering, injury prevention, aging and health problems, spine fatigue limitation frequency, lumbar degenerative disease, public health, left-behind elderly

## Abstract

**Background:** An increasing number of Chinese elderly women stay at home and act as grandchildren sitters. In consequence of the frequent load-bearing, chronic lumbar fatigue probably caused a higher risk of lumbar degeneration, fatigue, and injury which has become one of the most important aging and health problems in China. In this study, a multi-mode lumbar finite element model (FEM) with specific bone mineral density (BMD) were developed and validated for further spine injury prevention and control.

**Methods:** The material properties of lumbar vertebra were modified according to degenerated bone mineral density, and geometry was adjusted based on intervertebral disc height. The motion of lifting children was simulated by a 76 year-old Chinese women's FEM, and the stress distribution was calculated and predicted.

**Results:** The pressure of L5-S intervertebral disc in the bending 3-year-old dummy lifting posture was significantly higher than the same posture without lifting, the maximum effective stress of endplate cartilage in the upright child lifting posture was 1.6 times that of the bending without lifting posture. And the fatigue risk limitation frequency of the upright with dummy posture was predicted with the functional equation of fatigue and stress which was deduced by genetic algorithm, which combined with the effective stress of lumbar vertebrae spongy bone calculated from FEM.

**Conclusions:** The child-lifting motion could increase the risk of lumbar degeneration, fatigue, and injury in elderly women, and they should keep below the frequency limit of the motion of lifting children in their daily life. This study could put forward scientific injury prevention guidance to Chinese elderly women who lift children in daily life frequently.

## Introduction

Grandparent-raising refers to the situation where children are raised up by grandparents alone, or mainly by grandparents while parental rearing only plays a minor role. This phenomenon has attracted more and more investigators' attention (1–5). Grandparent-raising is influenced by cultural differences and mainly exists in East-Asian countries, especially in China. Because traditional culture in these countries emphasizes family harmony and collective happiness in the whole family, it is a common phenomenon that children are taken care of by their grandparents instead of their parents (1, 5, 6). Moreover, in these families, it is usually the responsibility of women to take care of children (5). During the course of babysitting, the action of lifting children, which involves bending, flexing, and the upright motion of the lumbar region, is inevitable and repeated frequently. In the view of biomechanics, those movements increased load-bearing on the lumbar vertebra and soft tissues. Because of the characteristics of the lumbar vertebra in elderly women, such daily lifting behavior is likely to increase the risk of lumbar fatigue (7, 8). With severe population aging and baby booming caused by the implementation of the second-child policy in China, more and more elderly women are at the risk of lumbar fatigue due to such daily behavior.

Different from injury of other parts of the human body (e.g., intervertebral disc protrusion) (9), lumbar fatigue, also called the silent epidemic, is usually asymptomatic and is easily neglected by the public (10). Most lumbar vertebral fractures are caused by common actions in daily life (8), movements like bending and lifting light objects are likely to increase the risk of lumbar vertebral fractures (7). Osteoporotic vertebral fracture is common in the elderly, representing a serious event, causing reduced activity or bedridden status with high mortality and morbidity rates, imposing a heavy burden on public health and social development (11). Due to the high misdiagnosis rate of this silent epidemic in clinical imaging examination (12–15), most elderly women with minor lumbar problems fail to get the doctors' warning and still frequently perform movements of bending, flexing, and lifting motions in raising their grandchildren, which leaves a latent danger of further serious damage. Therefore, it is crucial to predict and quantitatively evaluate the risk of such daily movements before serious lumbar injury occurs.

Some research efforts have been focused on applying the finite element model (FEM) to predict the degenerative disorder of lumbar/cervical spine and detect lumbar stress during operations (16–18). In addition, the use of the genetic algorithms in the quantitative evaluation of the body's tolerability to injury has made significant progress (19, 20). Based on these improvements, our study first developed a FEM based on two main characteristics of lumbar spine in elderly women, i.e., the decreased bone mineral density (BMD) and the reduced intervertebral space. Secondly, based on the measured result of the lumbar activity range of volunteering experiment subjects when they are lifting a dummy representative of a 3-year-old, we used FEM to set the range of motion and then calculated and predicted the muscle force and effective stress response of the intravertebrae disc, endplate cartilage in bending without lifting, bending with lifting a 3-year-old child, and upright with lifting 3-year-old child respectively. Thirdly, according to the fatigue-frequency curve of the lumbar spongy bone (21), we used the genetic algorithm to predict the frequency limitation of bending and upright posture with lifting corresponding to the effective stress of vertebrae spongy bone, then proposed a frequency limitation of such daily lifting movement and provided a quantitative reference for lumbar protection in elderly women who get involved with daily grandparent raising.

## Methods

### Description of the Finite Element Model

**Figure 1:**
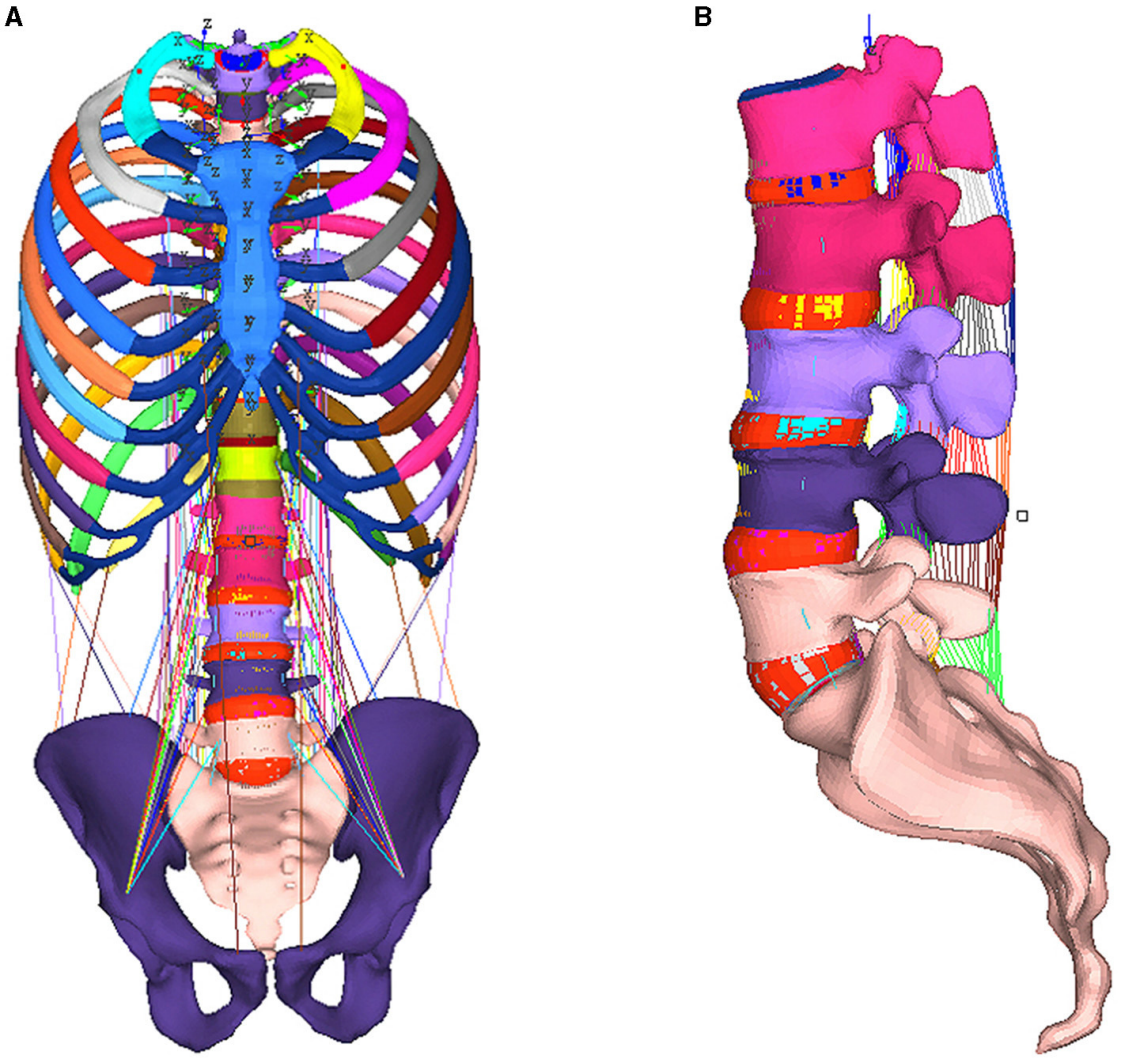
Integral map of lumbar spine model **(A)** and the lumbar spine FE model [**(B)** excluding muscle].

The FE model includes: vertebral body, intervertebral discs, main ligaments, and muscles (as shown in Figure 1A). Most anatomical geometry of the model comes from the CT scanning of 75-year-old women without clinical symptoms of lumbar degeneration. Lamellar thickness is 0.5 mm and resolution is 512 × 512. Then MIMICS software (Version 12.0, Materialize Inc., Leuven, Belgium) was used for the three-dimensional reconstruction. The intervertebral disc, endplate cartilage, and ligament were reconstructed according to anatomical position and reference from the radiologist. In order to make the FE model more consistent with the morphological characteristics of the target population, we adjusted the intervertebral space according to Shao's study (22). Elastic modulus of cancellous bone was adjusted according to the BMD of Chinese women population in Wu's report (23). The BMD was calculated based on equation (1)


(1) $$\begin{array}{l} BMD(age)=0.317+0.486•age-0.0011•age^{2}\\ \;+0.0000066•age^{3} \end{array}$$

Herein age was 60, and young's modulus was calculated by equation (2)


(2) $$\begin{array}{r} E=24∙BMD-3.73 \end{array}$$

Young's modulus of vertebrae spongy bone for Chinese elderly women was obtained, as shown in Appendix Data 1.

### Validation of the Lumbar FE Model Without Muscle

Since there is not available cadaver experimental data of the muscle for validation, in order to ensure the biofidelity of our model, we removed the muscle, then the lumbar FE model was validated by comparing its predictions with range of motion (ROM) observed *in vitro* under the flexion (8 Nm), extension (6 Nm) loading condition without preloading (24). The same as the description of the experiment *in vitro*, the boundary condition of the lumbar FE model without muscle was set identically with experiments *in vitro*, as shown in Figure 1B.

### Muscle Model Setting (Muscle Function Description)

In order to simulate the movement of an elderly woman in the process of lifting a child, the main muscles that maintain the stability of the trunk and waist when load-bearing were added into the model: Erector spinae, rectus abdominis, internal oblique muscle, external oblique muscle, lumbar major muscle, quadratus lumborum and multifidus. The psoas, multifidus, and quadratus lumborum muscle groups are reported to act as stabilizers of the lumbar spine. The erector spinae and the abdominal muscles are the primary locomotors of the spine. The parameters of the material properties are in the attachment (I–II), referring to the findings of Christophy (25). We referred to spine surgeons in Xiangya 3rd hospital when deciding the muscle starting and stopping points. The material parameters were converted according to PCSA (physiological cross-sectional area) and maximal force with equation (3), herein σ was the maximum engineering stress, *F*_max_ was the maximum force, the PCSA was the physiological cross-sectional area.


(3) $$\begin{array}{r} \sigma =\frac{F_{\max}}{PCSA} \end{array}$$

These variables were utilized as parameters of the fiber element parameter in LS-DYNA (LS-DYNA3D 971, LSTC, Livermore, CA, USA), as shown in Appendix II, and calculated in finite element model simulations. Similar settings were used to simulate the neck muscle response in the front impact study by Matthew's group (26).

### Volunteer Experiment and the Simulation of an Elderly Woman Lifting a Child

**Figure 2:**
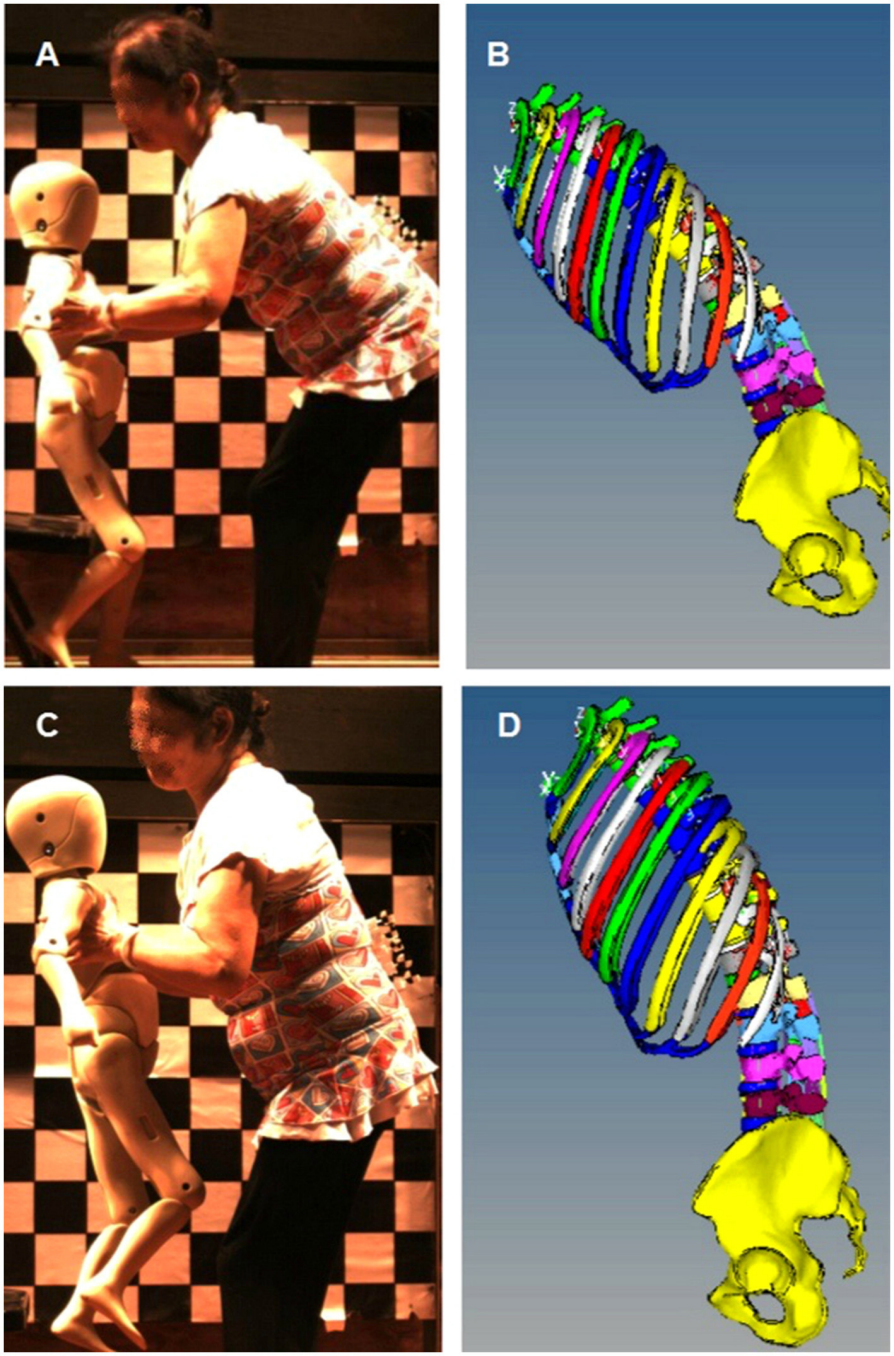
FEM mechanical simulation of volunteers lifting dummy. **(A)** Bending posture with lifting 3-year-old dummy, **(B)** Lumbar FEM adjusted posture according **(A,C)** Upright posture with lifting 3-year-old dummy, **(D)** Lumbar FEM adjusted posture according **(C)**.

In order to simulate the motion trajectory of an elderly woman lifting a child, a high-speed camera imaging system (Redlake MotionXtra HG-LE, DEL Imaging Systems, LLC. Chesshire. CT, US) was used to obtain the lumbar spine track in the process of volunteers lifting a dummy representative of a 3-year-old, as shown in Figures 2A,C. A standard 3-year-old child model (weight 16 kg, height 115 cm, P3 child dummy, Hunan SAF Automobile technology Co. Ltd, China) was used in this experiment. After labeling on the lumbar spine of the volunteers (Labeling spot was located on Spinous), video with a speed of 200 frames per second was recorded. (The characteristics of the volunteers: Age 76, 159.3 cm, 60.4kg, which is near the approximate the median of Chinese elderly women). The posture of the FE model was adjusted, as shown in Figures 2B,D, the range of motion of lumbar FE model was set identically to the volunteer's experiments. Three postures were simulated, the stress of the lumbar spine was calculated and predicted under bending without lifting posture, bending with lifting 3-year-old child posture, and upright with lifting 3-year-old child posture, respectively.

### Fatigue Curve

Repetitive loading lower than ultimate loads may cause failure (21), which presents a certain functional relationship between the action frequency and maximum stress. In this study, an equation was deduced from low cycle and high cycle fatigue studies with a genetic algorithm, as shown in equation (4). Herein σ_*eff*_ was effective stress, the unit was kPa, *N* was the number of cycles to failure.


(4) $$\begin{array}{r} N=1525-3.38∙\sigma_{eff}^{0.56} \end{array}$$

Based on the effective stress of vertebral spongy bone calculated from the 2.4 section, the combined genetic algorithm and the equation (4), our study quantitatively predicted the limited frequency of the lumbar vertebral spongy bone during the movement of elderly women lifting children.

## Results

### ROM Results Compared to *in vitro* Experiments

**Figure 3:**
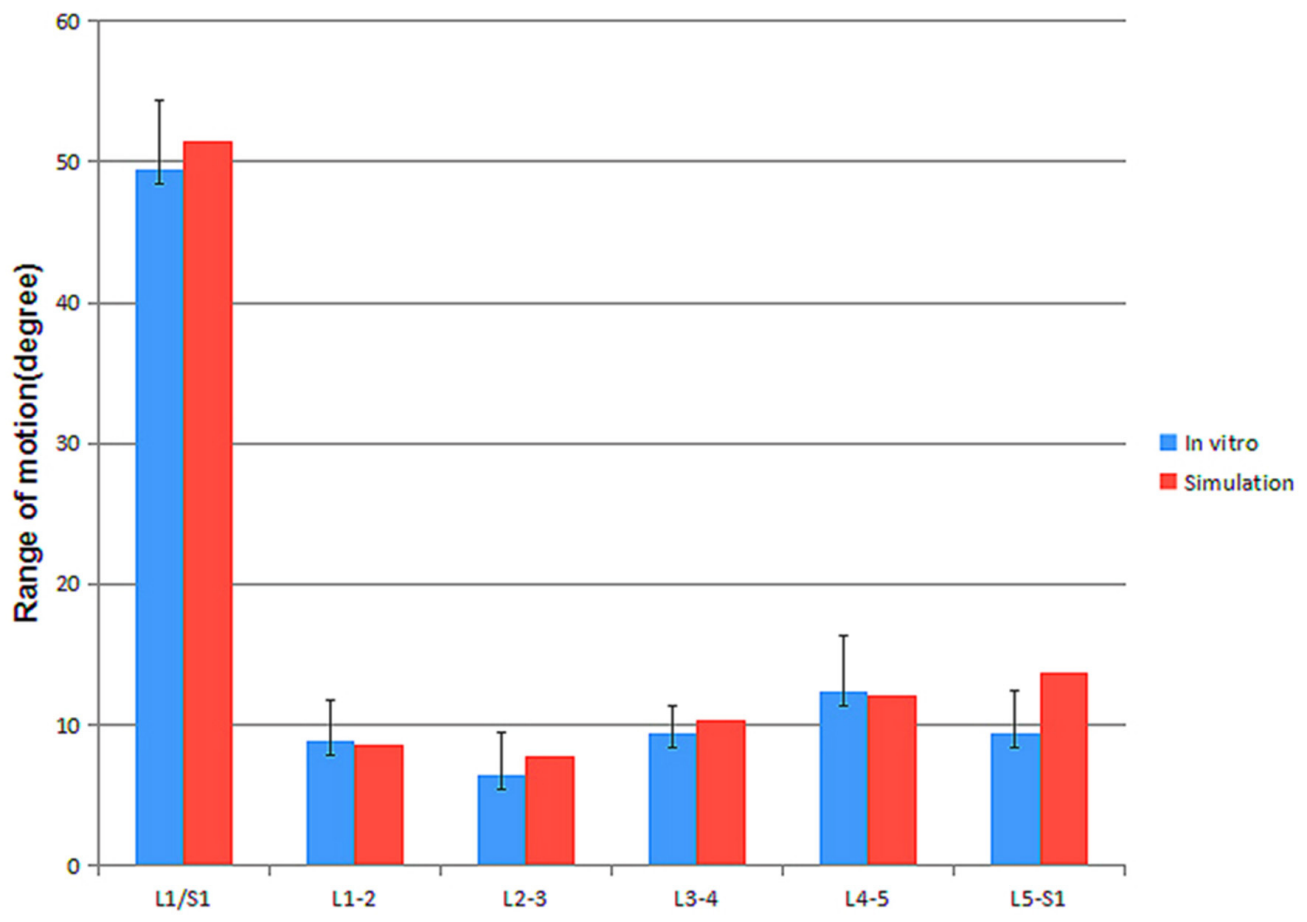
FEM simulation compared to the *in vitro* experiments data.

As shown in Figure 3, the ROM predicted by the lumbar FEM were in good agreement with the *in vitro* results at all segmental levels (24), except the L5-S1 ROM predicted was 13.7°, which was slightly above the *in vitro* result 9.43 ± .5°.

### FE Model Prediction of the Soft Tissue in Bending, Bending With 3-Year-Old Baby, and Upright With 3-Year-Old Child Posture

**Figure 4:**
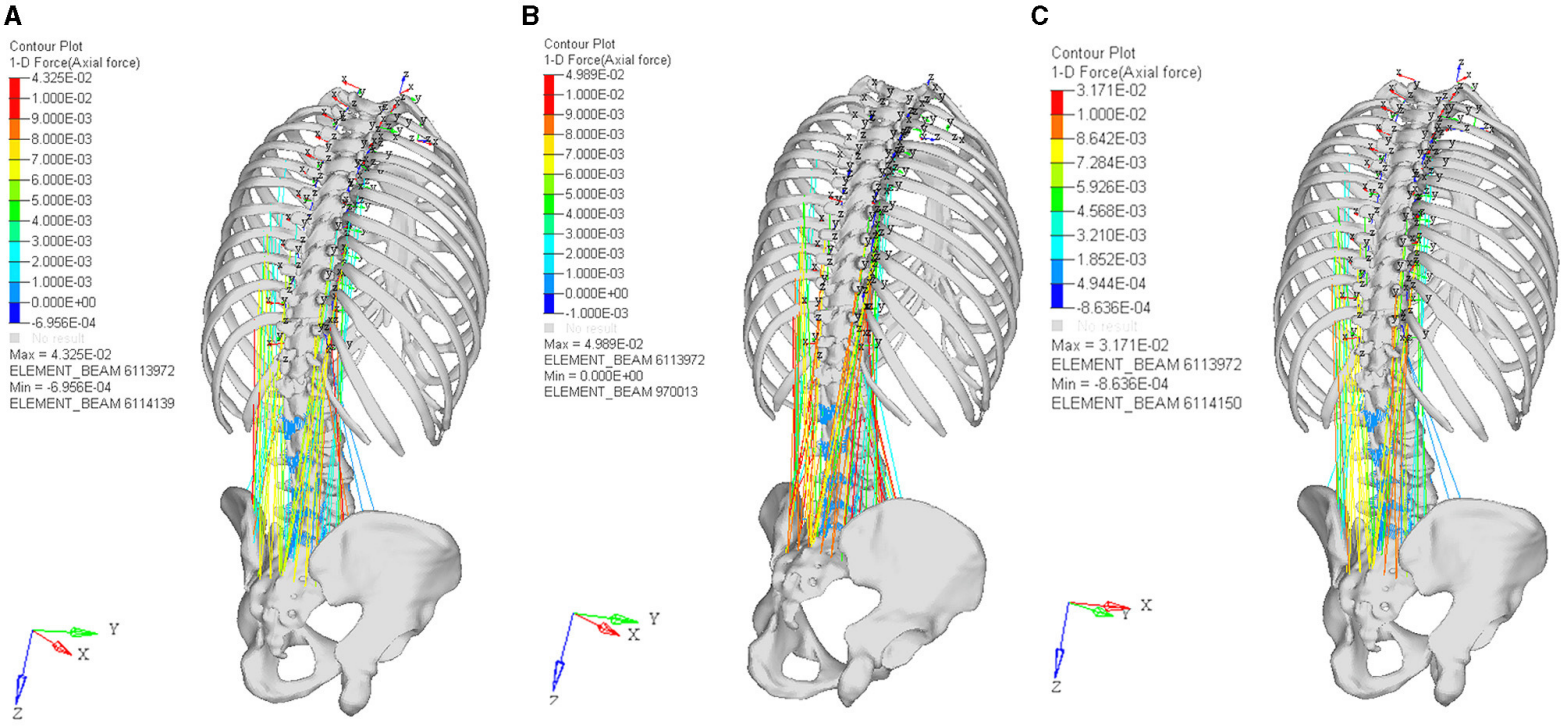
The muscle force contribution of bending without lifting weight posture **(A)**, the bending with lifting 3-year-old child posture **(B)**, and upright with 3-year-old child posture **(C)**.

**Figure 5:**
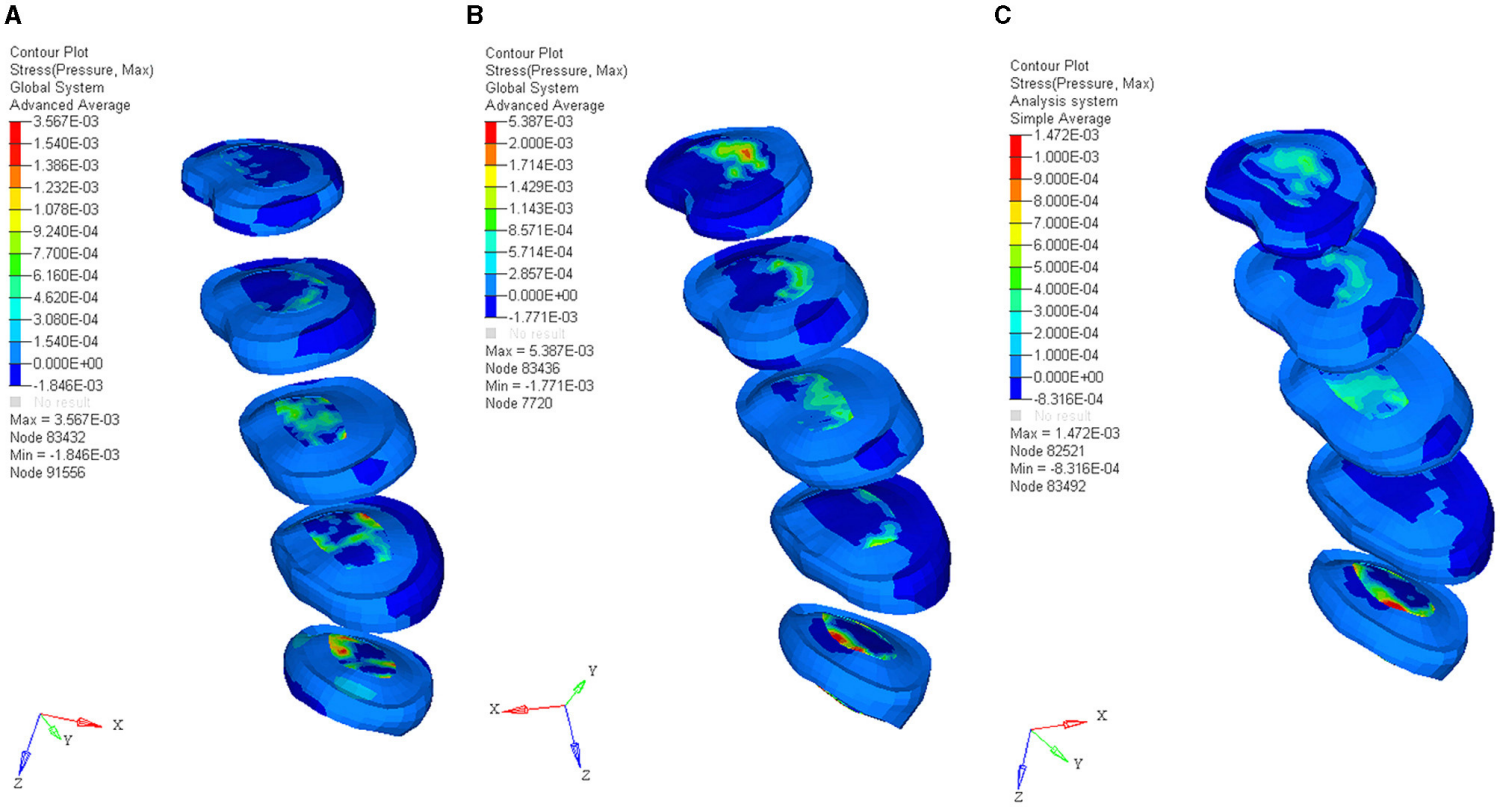
The maximum intradiscal pressure of bending without lifting weight posture **(A)**, the bending with lifting 3-year-old child posture **(B)**, and upright with 3-year-old child posture **(C)**.

**Figure 6:**
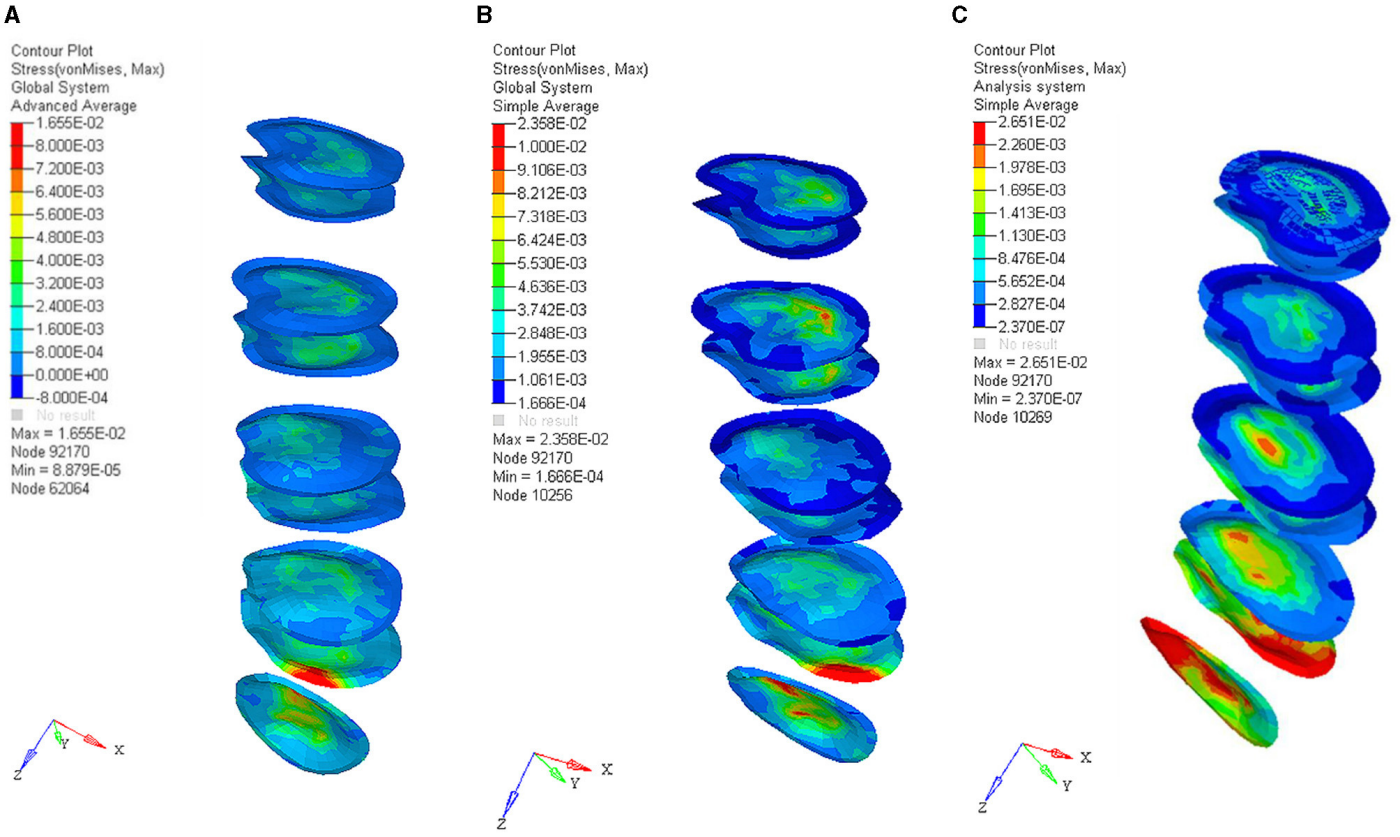
The effective stress of endplate cartilage in bending without lifting weight posture **(A)**, the bending with lifting 3-year-old child posture **(B)**, and upright with 3-year-old child posture **(C)**.

The experimental result showed that the maximum erector spinae tension was 4.32 kN, 4.9 kN, and 3.7 kN, in the bending without lifting weight, the bending with lifting 3-year-old child, and upright with 3-year-old child posture respectively, as shown in Figure 4. The calculated maximum intradiscal pressure was located in L5-S, which were 3.56 Mpa, 5.38 Mpa, and 1.47 Mpa, in the bending without lifting weight, the bending with lifting 3-year-old child, and upright with 3-year-old child posture respectively, as shown in Figure 5. The maximum effective stress contribution of endplate cartilage was located in posterior edge of L5 up and down endplate cartilage, which were 16.55 Mpa, 23.58 Mpa, and 26.55 Mpa in the bending without lifting weight, the bending with lifting 3-year-old child, and upright with 3-year-old child posture respectively, as shown in Figure 6.

**Figure 7:**
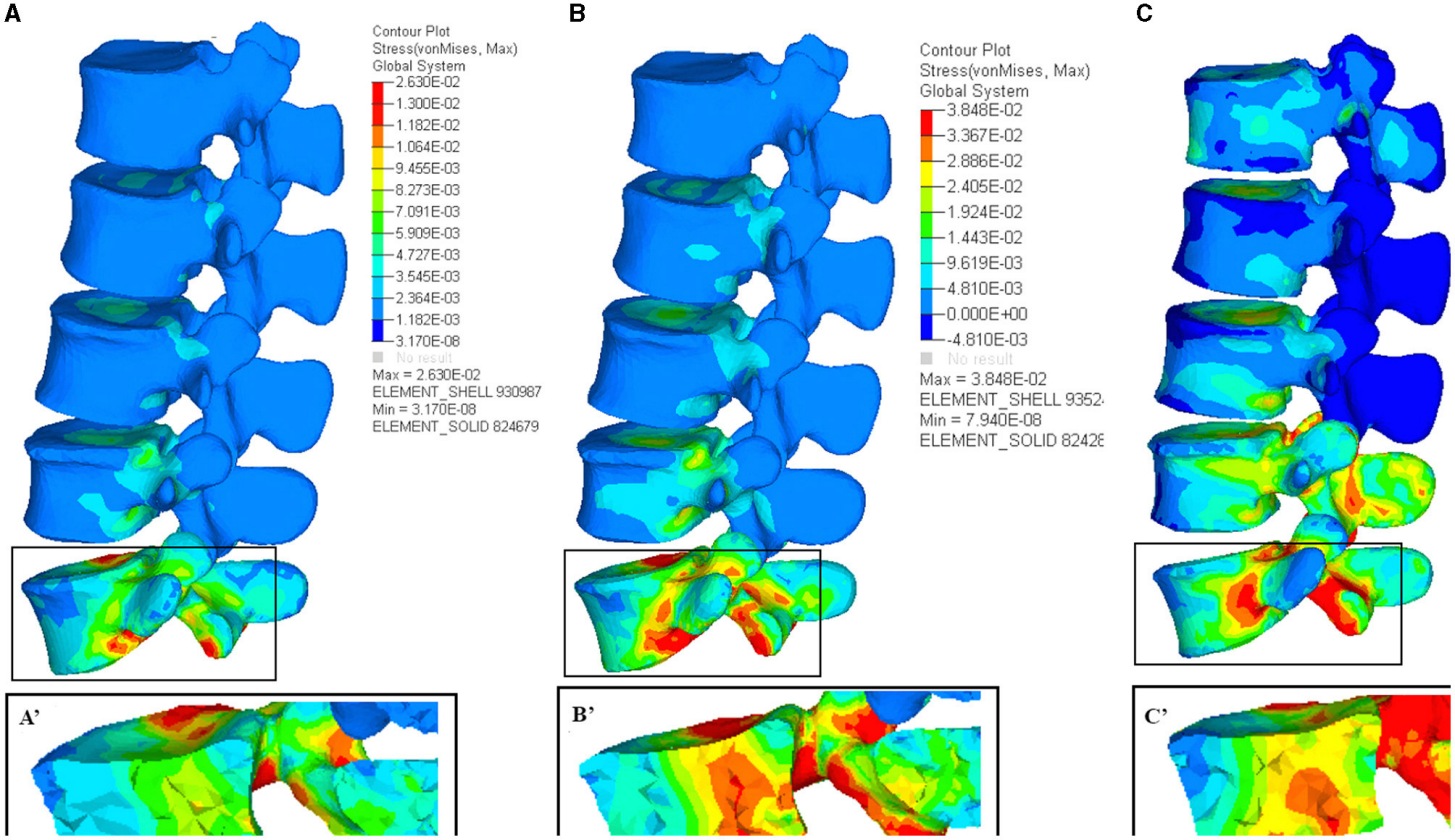
The vertebrae effective stress contribution in bending without lifting weight posture **(A)**, the bending with lifting 3-year-old child posture **(B)**, and upright with 3-year-old child posture **(C)**, the A', B', and C' were the detailed sagittal view of spongy bone of **(A–C)** respectively.

The detailed sagittal views of the effective stress in lumbar vertebrae spongy bone were shown in Figure 7, as the vertebrae spongy bone was the primary compression-bearing structure. In the bending without lifting posture, the maximum effective stress of vertebrae spongy bone was located in the lateral posterior part and the vertebral pedicle of L5, which was 26.3 Mpa. When the 3-year-old child was lifted, the maximum effective stress contribution of the vertebrae spongy bone was moved into the middle posterior part and vertebral pedicle of L5, which was 38.5 Mpa, an increase of 46.3% compared to the bending without lifting weight posture. More increments occurred in the upright with 3-year-old child posture, the maximum effective stress contribution of the vertebrae spongy bone was moved into the bottom part and vertebral pedicle of L5, which was 53.8 Mpa, an increase of 39.7% compared to the bending with lifting posture. In spongy bone, the cycle to failure was intimately correlated to the effective stress, according to the cycle of failure vs. and the stress curve, the cycle to failure of lumbar vertebrae spongy bone in three different postures were shown in Table 1.

**Table 1:** The corresponding cycle to failure of three postures were deduced from the effective stress according to the cycle number to failure vs. the stress curve.

**Posture**	**Maximum effective stress (Mpa)**	**Corresponding cycle to failure**
Bending without lifting weight posture	26.3	515
Bending with lifting 3-year-old child posture	38.5	275
Upright with 3-year-old child posture	53.8	18

## Discussion

Computer simulation could be used to better understand the mechanical and biology change *in vivo* by the prediction of the three-dimensional finite element method recently (27). To analyze the mechanical effect of the daily baby lifting motion on the Chinese elderly women, a lumbar finite element model with the unique geometry of Chinese elderly women was developed in this study. The muscle force, intradiscal pressure, and effective stress of endplate cartilage and vertebrae in three critical motion postures were calculated. In this section, we will assess mechanical change on the lumbar soft tissue degeneration and the risk of vertebrae spongy fatigue in the daily baby-lifting motion of Chinese elderly women, based on our predicted results and the previous reports.

### The Mechanical Change on Soft Tissue and Relevant Lumbar Degenerations

Before and after lifting, the maximum muscle force in bending posture has no significant change, the primary tense muscles were erector spinae, psoas major, quadratus lumborum, obliquus internus abdominis, obliqus externus abdominis, and rectus abdominis. After upright with lifting of 3-year-old baby, the muscular tension of erector spinae was relaxed, the maximum muscle force was decreased 24.5%. Ekholm measured that the erector spinae tension was 3.9 kN in lifting 12.8 kg with straight knees, and decreased 20.4% compared to lifting 12.8 kg with bending posture (28). Our muscle analysis result was in good agreement with the previous lumbar loading kinematic study, which will improve the biofidelity of this FE modeling and provide a more accurate prediction to muscle kinematic response analysis.

After lifting a 3-year-old child, the calculated intradiscal pressure was 1.51 times that of the bending without lifting posture, compared to after upright with 3-year-old child posture, the calculated intradiscal pressure in bending posture with the same loading was 2.38 times that of upright posture. As Sato measured the *in vivo* volunteer introdiscal pressure in different body postures, the different postures had a significant influence on intradiscal pressure, the intradiscal pressure in bending posture was reported to be 2.45 times that of the upright posture in the same loading (29). As the increased intradiscal pressure was considered as the primary impact factor of intervertebral disc degeneration in old age population (30). Collectively, the bending posture will cause the intradiscal pressure to increase, the increased intradiscal pressure will increase the risk of lumbar degeneration, typically the elderly women who frequently lift babies in a bending posture, this bending loading-bear posture might expedite the intravertebrae disc degeneration of the elder women.

In three different postures, the maximum effective stress of endplate cartilage is both located on the upper and lower endplates of L5. After lifting a 3-year-old child in a bending posture, the maximum effective stress of endplate cartilage is increased to 1.42 times that of the bending without lifting posture. More incrementally, upright with lifting 3-year-old child posture, was 1.6 times that of in the bending without lifting posture. As Adams reported, the repeated increase of endplate cartilage compression will cause the change of stress contribution in adjacent intravertebrae disc, and the annulus fibrosus cell appeared metabolically abnormal (31). Taken all together, these results suggested that the cyclic lifting motion of a 3-year-old child in daily life will aggravate the degeneration of intravertebrae disc.

### Fatigue Risk Caused by Mechanical Loading of Spongy Bone

Equivalent stress distribution of lumbar spongy bone: When the old woman is not lifting children, posterior vertebral pedicle of L4–L5 bears the largest force, which is 26.3 Mpa. When the old woman was bending and lifting a child, the effective stress on this part significantly increased by 44.3%. A larger increment occurred when the subject is standing upright after lifting a child and the effective stress reaches 53.8 Mpa. This is consistent with Pollintine's finding (32, 33). This change in stress suggests that when elderly women lift a child and finally reach the state of being upright, the pressure on posterior vertebral pedicle reaches its peak. After conferring to fatigue data (21), we found that in the case of bending without lifting, the fatigue limit is rather high, which is 515 times, so no obvious fatigue risk exists in this situation. The fatigue limitation frequency of the bending with lifting 3-year-old child posture is 275 times. Basically, a few risks of fatigue exist in this situation as well. In the case of upright with lifting a child, the corresponding fatigue limitation frequency of maximum load-bearing in the posterior vertebral body is low to 18 times. The result suggests that if the movements of upright and lifting a three-year-old child is done over 18 times continuously, the posterior vertebral body and pedicle spongy bone of L4–L5 will face the risk of fatigue failure.

However, it should be noted that the fatigue curves we referred to are based on *in vitro* experiments of cadaverous spongy bone. Nevertheless, the material properties of *in vivo* and *in vitro* spongy bone are different. Taylor's study found that spongy bone *in vivo* can self-repair the injury caused by stress fatigue (21). Thus it can be speculated that *in vivo* spongy bone has a higher fatigue tolerance than *in vitro* spongy bone. However, since there is no reference data about the relationship between the load-bearing of *in vivo* spongy bone and its fatigue, our calculation is based on the *in vitro* data. Therefore, the risk frequency (18 times) in this study can only be considered as a conservative reference value for the limiting frequency of the lifting child movement in elder women.

Actually, the lifting movement which our study focused on is called Knee Straight Lifting (KSL), the fracture risk of which is the highest among all common kinds of lifting movements (7). For Chinese women over 55 years old, lumbar loading is a high-risk factor for lumbar degeneration (11). Mechanical and biological factors, which were interconnected and amplified each other (34, 35), were considered the primary roles of lumbar degeneration. In this study, the lifting baby motion will accelerate the lumbar degeneration and increase the risk of fracture in the following ways: increasing intervertebral disc pressure, increasing endplate cartilage pressure, and fatigue failure of vertebral spongy bone due to the repeated loading-bear. Increasing bone strength and avoiding high-risk action are two approaches to prevent such risk. This study provides a reference for elderly women in China and East Asia, which is trying to avoid continuous straight-standing action in the daily movement of lifting children. In a word, the frequency of KSL movement especially with burden should not be too much. The experimental model and stress analysis fully considered several closely related factors: BMD degeneration (23), muscle movement (25), ligament material parameters, and patient-specific skeletal geometry of asymptomatic elderly women. The article aims to simulate the biomechanical characteristics of Chinese elderly women as accurately as possible and quantitatively analyzes the movement of lifting children, which happens frequently in Chinese elderly women. The study possesses following characteristics. First, we added the main muscles and ligaments of the waist into the model, which helps to simulate the dynamic process of lifting a child with higher biofidelity. Second, in the motion simulation, the model simulated the process of lifting a child according to the result of high-speed photography in volunteers' experiments. Thirdly, the fatigue property of vertebral spongy bone was taken into consideration. We combined the research result with fatigue curve and get the fatigue limit frequency of vertebral part bearing the most pressure and quantified the effect of such pressure on the vertebral body's fatigue failure.

## Conclusions

Although China started its transformation earlier than other socialist countries, it is still undergoing social changes. Especially after the three-child policy change, more children would be left with their grandparents who have to provide support for their 8-to-6-working son or daughter, which commonly happens in China. Moreover, it is usually the responsibility of women to take care of children. And now, China is facing new ramifications from the three-child policy. More and more elderly Chinese women who act as stay-home grandchildren sitters are difficult to bear for the increasing expenditure of medical costs. The reform of medical insurance system and the process of medical privatization in China have resulted in medical treatment prices which have increased the difficulties of plenty of elderly women's capability to access healthcare. This study obtained the frequency limit of lifting child movement in the case of lumbar degeneration and put forward scientific guidance to elderly Chinese women especially for those aged over 75 years. With the deterioration of the aging problem in China and the increasing number of newborns, our study has practical significance to protect the lumbar health of elderly women.

Risk factors and mechanisms of injury-related health problems are of the most stubborn but most easily neglected issues in older Chinese people. We do hope that the multi-modal risk analysis of lumbar degeneration, fatigue, and injury based on FEM/BMD could put forward scientific injury prevention guidance to elderly Chinese women who are lifting lift children in daily life frequently, not only providing evidence for improving the health status of elderly women but also raising the women's health standard and improve the ability to prevent injury, as well as further explore the factors affecting the injury-related health problems of the elderly in China, which will also make a positive contribution to healthy aging globally.

## Data Availability Statement

The original contributions presented in the study are included in the article/supplementary material, further inquiries can be directed to the corresponding author/s.

## Ethics Statement

Written informed consent was obtained from the individual(s) for the publication of any potentially identifiable images or data included in this article.

## Author Contributions

JS and NL contributed to the conceptualization of the study, formal analysis, and supervision. KX and XD contributed to the data curation and conceptualization. MC contributed the write-up and editing of the article. JS contributed to the supervision, conceptualization, data curation, and final version write-up. All authors have read and agreed to the published version of the manuscript.

## Funding

Funding was provided by Excellent Postdoctoral Program for Innovative Talent of Hunan (2020RC2015), the China Postdoctoral Science Foundation (2020TQ0364 and 2021M693564), and Natural Science Foundation of Hunan (2020JJ5865).

## Conflict of Interest

The authors declare that the research was conducted in the absence of any commercial or financial relationships that could be construed as a potential conflict of interest.

## Publisher's Note

All claims expressed in this article are solely those of the authors and do not necessarily represent those of their affiliated organizations, or those of the publisher, the editors and the reviewers. Any product that may be evaluated in this article, or claim that may be made by its manufacturer, is not guaranteed or endorsed by the publisher.
